# Long-term patterns of forearm asymmetry in females of three syntopic bat species and its effects on individual fitness

**DOI:** 10.1038/s41598-024-80130-w

**Published:** 2024-11-20

**Authors:** Tobias Süess, Gerald Kerth

**Affiliations:** Applied Zoology and Nature Conservation, Zoological Institute and Museum, University of Greifswald, Greifswald, Germany

**Keywords:** Chiroptera, Climate change, Fluctuating asymmetry, Forearm length, Long-term data, Woodland, Ecology, Behavioural ecology, Conservation biology, Evolutionary ecology, Forest ecology, Evolution, Sexual selection, Zoology, Animal behaviour

## Abstract

Fluctuating asymmetry, the non-directional deviation from bilateral symmetry resulting from developmental instability, can indicate early-life environmental stress. While fluctuating asymmetry can affect individual survival and reproductive success, its effect on fitness differs between species. Here, we analyzed up to 27 years of mark-recapture data from 894 RFID tagged individuals of three forest-living bat species in southern Germany to investigate the degree of fluctuating asymmetry in forearm length. In Bechstein’s bats, *Myotis bechsteinii*, the species with the highest sample size, we furthermore investigated if fluctuating asymmetry has become more frequent over the study period, a time when juvenile bats have grown larger forearms in response to warmer summers. We also investigated whether fluctuating asymmetry affects individual lifespan and lifetime reproductive success in female *Myotis bechsteinii*. The degree of fluctuating asymmetry clearly exceeding the measurement error estimated on recaptured individuals was similar in all three species (1.8%). In female *Myotis bechsteinii*, the frequency of fluctuating asymmetry did not increase over the course of the study and even strong asymmetry had no effect on individual reproductive success and life expectancy. Our data suggest that fluctuating asymmetry is a poor predictor of fitness in the female *Myotis bechsteinii* studied, and is so far unaffected by the warming environment which is leading to larger individuals in our study population.

## Introduction

Fluctuating asymmetry (FA) describes the non-directional deviation from bilateral morphological characters^1,2^ that results from developmental instability, which translates into phenotypic features across taxa and traits (e.g., the central nervous system of vertebrates^3^; Cetacean skulls^4^; snake scalation^5^; flower morphology^6^). FA can be caused by various factors, including genomic and environmental stressors as well as trait history and is known to affect survival, reproductive success and offspring health^2,7–10^. There are two other types of asymmetry, namely directional asymmetry and antisymmetry^1^. Directional asymmetry describes bilateral morphological traits that are consistently larger on one side leading to mean asymmetry values that either are negative or positive. Antisymmetry also describes bilateral morphological traits that are larger on one side. However, in contrast to directional asymmetry, the larger side varies between individuals leading to bimodal or platykurtic distributions^11^.

In several bird and mammal species, environmental stressors, such as habitat fragmentation, positively correlate with FA. Anciães and Marini^12^ compared 100 tropical passerine birds in fragmented and continuous habitat and demonstrated that wing and tarsus asymmetry positively correlates with habitat fragmentation. A meta-analysis study of small mammals compiled data from studies spanning four decades and showed that environmental stress increased FA across traits in 70% of the studies used^13^ and references therein]. However, in some species no link between habitat quality and asymmetry has been shown. For example, Gebremichael et al.^14^ reported FA of tarsus length of 64 passerine bird species being similar in shaded coffee plantations and natural forests. Similarly, Coda et al.^15^ found no difference in FA of three rodent species in organic compared to conventional farming systems. In addition, habitat disturbance can also have opposite effects on FA between closely related species as in the two rodent species *Peromyscus maniculatus* and *P*. *leucopus*^16^. Overall, environmental stress has complex and multi-faceted effects on individual FA.

Trait history and sex can further modulate differences in FA^17,18^. Traits that increase rather than decrease through evolution show less efficient buffering against stressors and consequently, increased FA. In certain instances, the type of asymmetry is sex specific. For example, female common vampire bats (*Desmodus rotundus*) show FA, whereas male vampire bats show directional asymmetry and antisymmetry^19^. Similarly, males in great grey shrikes (*Lanius excubitor)* show directional asymmetry in contrast to females^18^.

FA can impair movement, health, survival, and lifetime reproductive success^5,7,10,20–25^. However, FA sometimes has no effect on fitness and reproductive success, such as in red-winged blackbirds (*Agelaius phoeniceus*)^26,27^. Cuervo et al.^23^ studied three gazelle species and found evidence for unhealthier blood parameters (platelets, albumin and lactate hydrogenase) in more asymmetric individuals. Hindleg asymmetry of wood mice (*Apodemus sp*.) was positively correlated with predation risk by tawny owls^24^. Asymmetric male garter snakes (*Thamnophis sirtalis parietalis)* were less able to secure matings than symmetric males^5^. In the neotropical Seba’s short-tailed bat *Carollia perspicillata*, asymmetry of forearms decreases the probability of having two pregnancies per year^10^. Likewise, in the neotropical sac-winged bat *Saccopteryx bilineata* symmetric but also smaller males had a higher reproductive success than asymmetric and larger males^25^. In contrast, using five bilaterally paired morphological traits, Dufour and Weatherhead^26,27^ showed that asymmetry had no effect on male reproductive success (e.g., harem size, breeding habitat quality, reproductive skew and reproductive success of female mates) in *Agelaius phoeniceus*.

Symmetry in one feature may offset asymmetry in other traits, depending on the importance of the trait, potentially resulting in no fitness effects from FA. For example, asymmetry of one wing feature is compensated for by other wing features in *Desmodus rotundus*^19^. In bats in general, traits that are functionally more important for larger movement distances, such as forearms, are less asymmetric than legs (tibia,^28^). Similarly, the hindlimbs of rabbits, used for jumping, show lower levels of asymmetry than the forelimbs^29^.

Here, we analyzed 5142 forearm length measurements, including repeated measures over multiple capture events, on a total of 894 female bats. These data originated from up to 27 years of monitoring three syntopic forest-living bat species (*Myotis bechsteinii, M. nattereri, and Plecotus auritus*) from Germany. In two of these species, *Myotis bechsteinii* and *Myotis nattereri*, forearm length increased in recent years, due to warmer conditions during early life^30,31^. While this increase leads to a faster pace of life in *M. bechsteinii*, larger females have a similar lifetime reproductive success because of an earlier onset of reproduction. In *M. nattereri.*, larger females even have a fitness advantage^31^. In both species, FA has been occasionally reported before^32,33^, however, it is unknown whether asymmetry in forearm length has fitness consequences in any of our three study species. Moreover, it is unknown whether FA is affected by the observed recent increase in body size in *M. bechsteinii*, which could be indicative of changed environmental stress levels at times of increasing ambient temperatures.

Based on the results of the previous studies on FA in different taxa, we predicted that strong female forearm length asymmetry reduces lifespan and lifetime reproductive success^10,25^. Moreover, we predicted that FA occurs at a similarly low rate in the three studied German bat species because of the importance of forearms for flight^28,29^. Finally, we assessed whether FA changed over the course of the study, a period the average forearm length increased in *M. bechsteinii* and *M. nattereri* due to warmer temperatures during the postnatal development of the juveniles^31,32^.

## Results

**Fig. 1:**
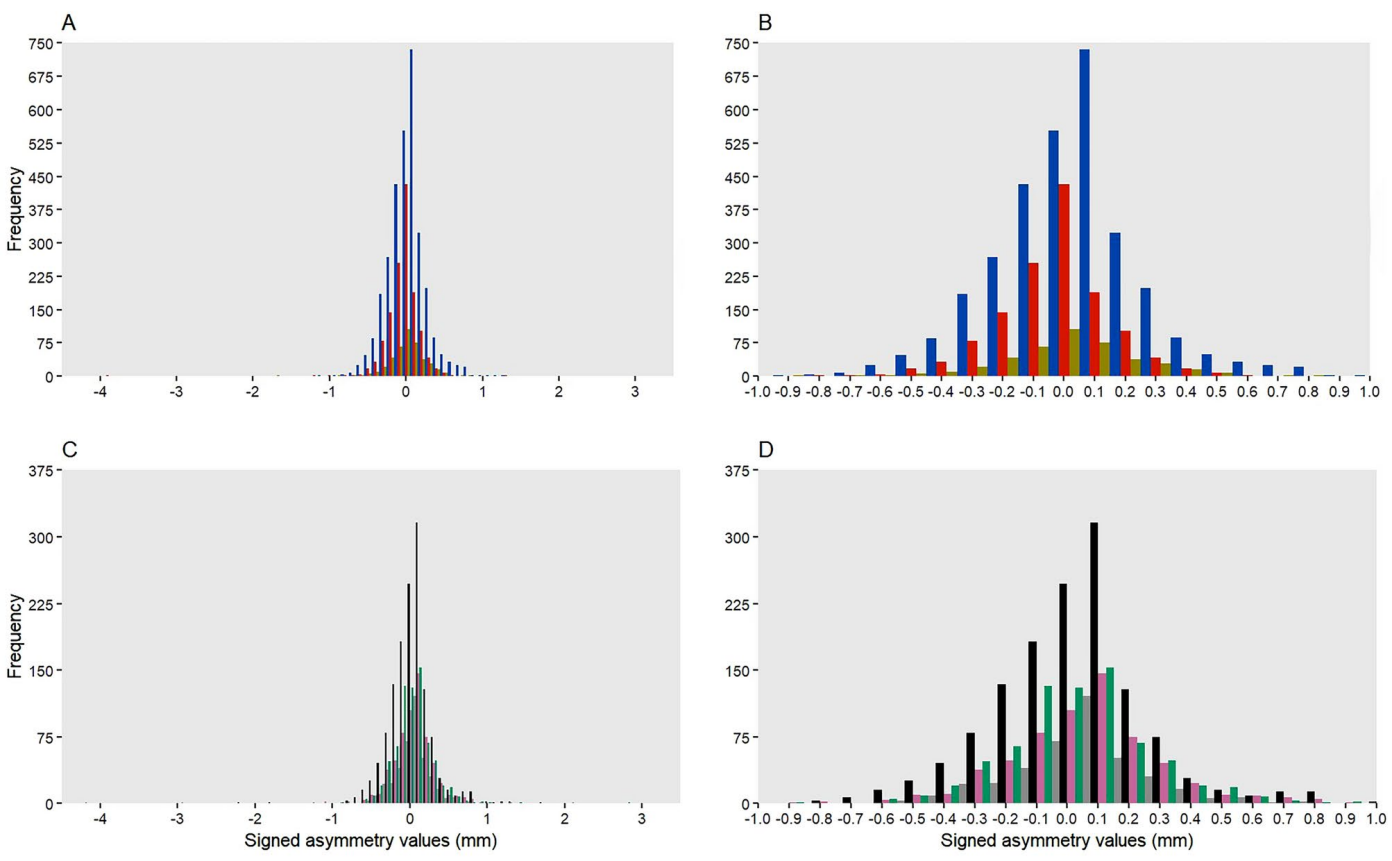
Distribution of female forearm length asymmetry (mm). (**A**) *M. bechsteinii* (blue), *M. nattereri* (red) and *P. auritus* (yellow) wide and (**B**) narrow and (**C**) *M. bechsteinii* in colony BS (grey), GB2 (black), HB (pink) and UA (green) wide and (**D**) narrow**.**

**Fig. 2:**
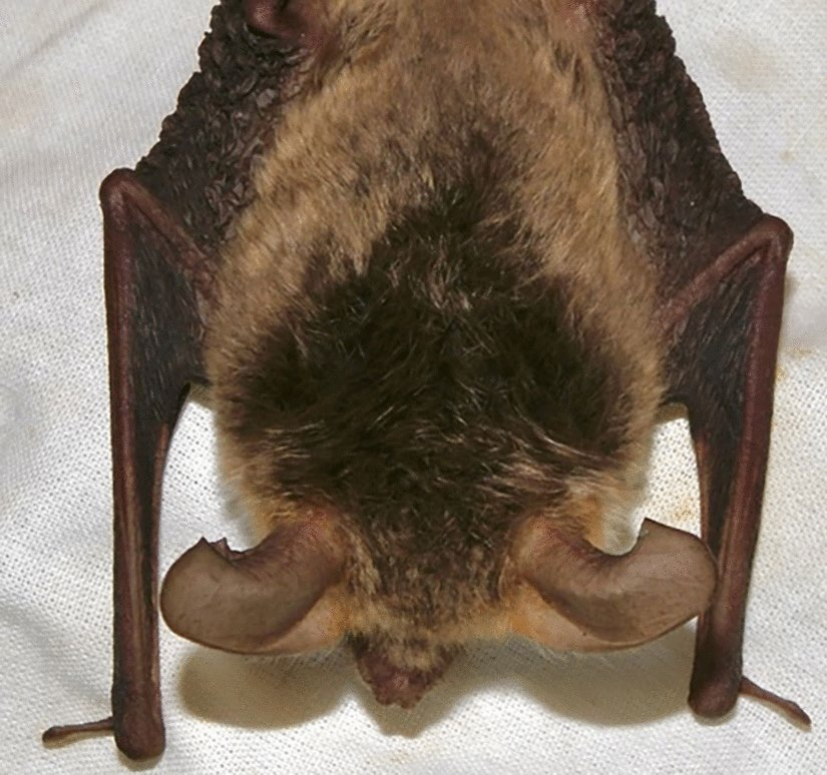
Picture of highly asymmetric forearms in a female *M. bechsteinii* (ID = 672). This shows the visible forearm asymmetry of *M. bechsteinii*.

**Fig. 3:**
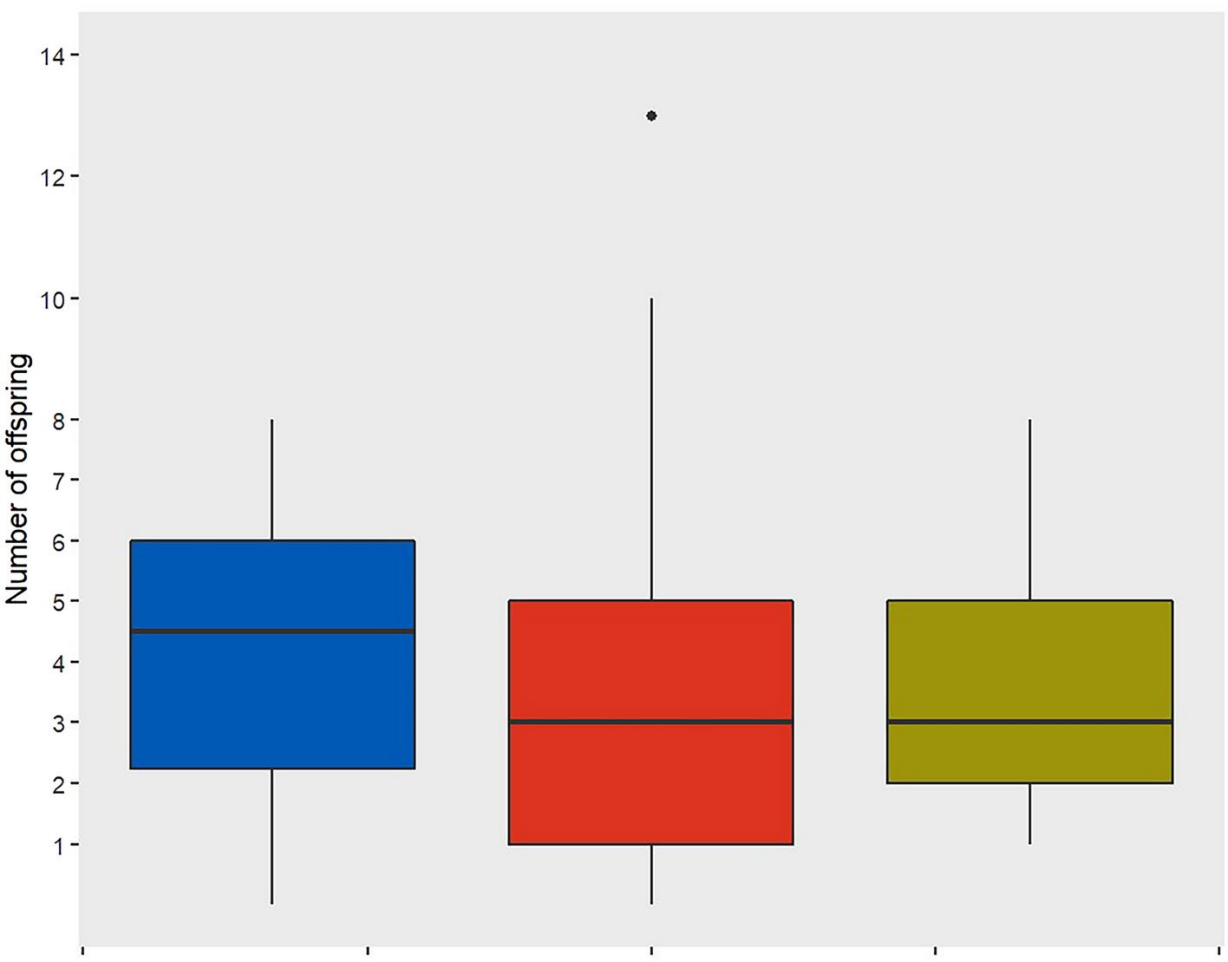
Clearly asymmetric females of *M. bechsteinii* (blue) have a similar number of offspring compared to clearly symmetric females (red) and females with unknown forearm symmetry (yellow). The boxes represent median and 25%/75% quartiles, with whiskers extending 1.5 × beyond interquartile range and outliers as black squares.

The measurement error exceeded the caliper’s precision by 122—230% (Tables 1 and 2). The distribution of the asymmetry values shows leptokurtic distributions in all studied species (Fig. 1). The mean forearm length (+ SD) of females was 42.72 ± 1.11 mm for *M. bechsteinii*, 40.48 ± 1.09 mm for *M. nattereri* and 40.22 ± 1.08 mm for *P. auritus*. The number of measurements had no effect on the extent or direction of asymmetry (p = 0.182). All observers had on average similar values for all measurements on the right forearm compared to all measurements on the left forearm (all p-values > 0.98). Females of all three studied species exhibit fluctuating and directional asymmetry, but no antisymmetry. The median [interquartile range] asymmetry of forearm lengths was similar in all species (*M. bechsteinii* = 0.1 [0.1–0.3] mm, *M. nattereri* = 0.1 [0–0.2] mm, *P. auritus* = 0.1 [0.1–0.3] mm, Table S1). Of all caught adult female bats, 1.8% are clearly asymmetric (more than 0.3 mm difference in left and right forearm length). Two individuals showed very pronounced asymmetry. Firstly, one *M. nattereri* (ID = 1536), which was first captured in 2013, shows a mean asymmetry of 3.84 mm (range between repeated measures: 3.5–4.1 mm) and reaches at least five years. The most asymmetric individual, a *M. bechsteinii* (ID = 672) first captured in 2007, shows a mean asymmetry of 5.38 mm (range between repeated measures: 4.3–5.8 mm) and reaches at least 10 years (Fig. 2). Asymmetric *M. bechsteinii* (asymmetry ≥ 0.3 mm) had a similar number of offspring (n = 12, mean = 4 ± 3, p = 0.26, Table S1, Fig. 3) as symmetric individuals (every measurement with a maximum asymmetry ≤ 0.1, n = 37, mean ± SD = 3 ± 2). Birth year but not asymmetry affected reproductive success and lifespan (Tables S1, S2). *M. bechsteinii* that were born in 2011, 2013 and 2017 had significantly fewer offspring during their lifetime than bats born in other years. Conversely, *M. bechsteinii* that were born in 2008, 2009 and from 2016–2019 lived significantly shorter lives than bats born in other years. Furthermore, *M. bechsteinii* from colonies GB2 and UA have significantly longer lifespans than from colony BS (BS, 4 [3–7] years; GB2, 5.0 [3–9] years; UA, 4.5 [2–6] years; Table S2). Neither bat species nor birth year affected asymmetry (Table S2).

**Table 1:** *M. bechsteinii* have similar levels of fluctuating asymmetry irrespective of colony and birth year (Gamma).

Fixed effect	Sample size	Estimate	SE	p-value
Intercept		6.05	0.51	< 0.001
Site GB2	196	-0.18	0.41	0.66
Site HB	143	-0.67	0.44	0.12
Site UA	71	-0.22	0.45	0.63
Birth Year		0	0.02	0.8

Random effect	Variance	SD		
Individual	3.07	1.75		
Observer	0.05	0.22

**Table 2:** Fluctuating asymmetry is present in all three species (two-factor mixed ANOVA).

*M. bechsteinii*	ANOVA				
	df	Sum Sq	Mean Sq	F	p-value
Side	1	0.400	0.421	3.671	0.055
Side:individual	517	195.000	0.377	4.247	< 0.001
Side:observer	11	732.000	66.550	5.091	< 0.001
Residuals	5701	654.500	0.115		
Measurement error			0.339		
Excess kurtosis	Β2–3 = 83.08	P = < 0.001			
*M. nattereri*					
Side	1	0.070	0.072	1.295	0.255
Side:Individual	267	65.240	0.244	7.674	< 0.001
Side:Observer	9	95.300	10.580	0.897	0.529
Residuals	2410	133.160	0.055		
Measurement error			0.235		
Excess kurtosis	Β2–3 = 73.06	P = < 0.001			
*P. auritus*					
Side	1	0.060	0.061	1.379	0.241
Side:Individual	125	13.594	0.109	3.414	< 0.001
Side:Observer	8	96.500	12.060	1.528	0.155
Residuals	740	32.770	0.044		
Measurement error			0.210		
Excess kurtosis	Β2–3 = 8.786	P = < 0.001

## Discussion

This comprehensive repeated-measures study spanning 27 years indicates that in the studied populations of three sympatric forest-living bat species, females show fluctuating and directional asymmetry in the forearm. After correcting for directional asymmetry, fluctuating asymmetry was relatively rare in the studied *M. bechsteinii* , *M. nattereri* and *P. auritus* (1.8%). In part, this result may have been affected by the conservative threshold we applied to avoid using asymmetry values within the mean measurement error. Our relatively high measurement error was due to measuring alive bats in the field. Despite these limitations, the observed low frequency of asymmetric individuals in the three study species was to be expected because of the importance of forearms for flight and is well in the range of reported FA in wing parameters in other studies bats^10,25^.

Interestingly, even strong forearm asymmetry (≥ 5% of the total forearm length) had no negative consequences on the survival and lifetime reproductive success of female *M. bechsteinii* the only species for which we had a sufficient sample size of strongly asymmetric individuals. While FA has been found to negatively affect fitness in a variety of species^7,20–22^, other studies, like ours, found no such effect. For example, Behr et al.^34^ found no link between asymmetry and reproductive success in the neotropical greater sac-winged bat (*Saccopteryx bilineata*). Dyrcz et al.^35^ showed that asymmetry of different wing features had no effect on the number of nestlings in aquatic warblers (*Acrocephalus paludicola*). This species had higher levels of FA compared to our studied species (mean = 0.75 mm), which corresponds to about 1.2% of their average wing length. European barn swallows (*Hirundo rustica*) had a lifetime reproductive success that was not influenced by the asymmetry of tail feather length^36^. For the same species, Cadée^37^ showed that wing asymmetry had no effect on offspring body conditions. However, the extent of wing asymmetry was pronounced (mean = 0.45 – 0.86 mm), but while the barn swallows are larger, the study didn’t provide the total wing length. Finally, in pied flycatchers (*Ficedula hypoleuca*), FA of various features, including flight feathers, had no effect on body condition, survival or reproductive success^38^.

Several causes could explain why even clearly asymmetric female *M. bechsteinii* had a similar lifetime reproductive success than symmetric females in our study population. Firstly, asymmetry of forearm lengths might have only minor effects on flight performance and thus survival, because other wing features might compensate for asymmetric forearms^19^. We could not address a possible compensation effect, as only data on forearm length were available. For comparison, we know of only one study that showed that fluctuating asymmetry leads to declining flight performances in European starlings^39^. Thirdly, asymmetry might only affect mortality in early life whereas our data set included only adult, fully grown females. Several species of the genus *Myotis*, namely *M. nattereri* and *M. daubentoni*, show a relatively high juvenile mortality^40,41^. In *M. bechsteinii* juvenile mortality is higher than in adults too^31^. Thus, it would be interesting to study whether asymmetry in forearm length might increase mortality in juveniles. FA might only affect the lifetime reproductive success of male *M. bechsteinii* through sexual selection and male-male competition during autumn swarming (*M. bechsteinii* mate during swarming in autumn in front of hibernacula^42^). In comparison, female *Saccopteryx bilineata* prefer symmetrical males to asymmetrical males^25^. Males of *Saccopteryx bilineata* had similar mean levels of forearm asymmetry, namely 0.10–0.12 mm, which corresponds to about 2% of their mean forearm length. Although *M. bechsteinii* shows a low reproductive skew^33^, highly asymmetric males might still have a lower lifetime reproductive success than symmetric males. However, this question could not be addressed in our study, because males were rarely recaptured as adults, as they disperse from their natal colony and live solitarily afterwards (43). Finally, we found no clear trend of declining or increasing FA over the years. Thus, while female *M. bechsteinii* grow larger forearms in warmer summers in recent years^30,43^ their FA did not change and remained low. This is in contrast to a comparative study on various bird species^44^ that found fluctuating wing asymmetry to increase in response to global warming.

In summary, in female *M. bechsteinii* for which we had data for an in-depth analysis, fluctuating asymmetry did not affect either lifespan or lifetime reproductive success. Therefore, fluctuating asymmetry in forearm length is a poor predictor of female individual fitness in the *M. bechsteinii* populations studied, despite the fact that absolute forearm length is negatively correlated with lifespan^45^.

## Methods

### Ethics declaration

Handling, tagging, and monitoring of the bat individuals are conducted under permits in accordance with the guidelines and regulations for species protection (55.1.-8642.01/00) and animal welfare (55.2-DMS 2532–2-20) issued by the government of Lower Franconia in Bavaria. The Veterinary Office of the government of Lower Franconia is the institutional committee that approved all experimental protocols. All methods are reported according to the ARRIVE guidelines.

### Study sites and capture methods

**Fig. 4:**
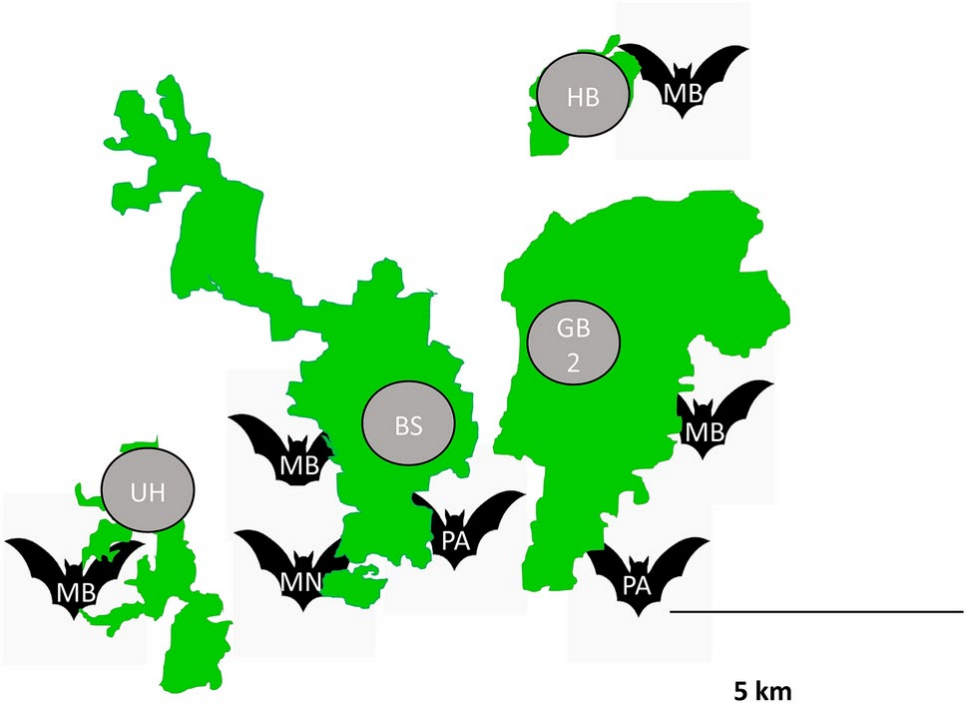
Map of the four study sites. *M. bechsteinii* (MB) is present at all study locations, but neither *M. nattereri* (MN) nor *P. auritus* (PA).

This study uses a long-term monitoring dataset of female bats ranging from 1996 (*M. bechsteinii*), 2008 (*P. auritus*), and 2011 (*M. nattereri*) until 2022. The bats where members of seven different colonies living in four deciduous forest sites (named BS, GB2, HB, UA; Fig. 4). All investigated bat colonies are situated near Würzburg, Germany, within 15 km of each other. The composition of tree species and levels of forest management are similar in the colonies’ home ranges. Bats were captured at least twice each year between May and September at their day roosts (bat boxes) and subcutaneously implanted with a distinct RFID-tag (RFID stands for Radio-Frequency Identification) for identification, if they lacked one^34^. The length of both right and the left forearms was measured with a caliper to the nearest 0.1 mm upon each capture. Fluctuating asymmetry was defined as the unsigned difference between the left and the right forearm L-R. Thanks to the continuous monitoring, we were able to calculate the true age of 84% of the captured bats and estimated the age of the remaining bats with an approximation of ± 1 year.

### Lifetime reproductive success

Lifetime reproductive success (LRS, total number of offspring successfully weaned in a female bat’s lifetime) was available from Mundinger et al.'s study^30^. LRS is only available for bats that were born and died within the study period.

### Statistical analyses

In all three species, bats reach adulthood and the forearm is fully developed one year after their birth. Therefore, we excluded all individuals less than one year old from our analysis, as their unfinished growth could bias our measurements. After one year, bats of all three species are fully grown. Furthermore, males were excluded from all downstream analyses due to small samples sizes of adult males (n_*M. bechsteini*_ = 40, n_*M. nattereri*_ = 19, n _*P. auritus*_ = 8) in all investigated bat species, due to male dispersal^46,47^. Unlike females, males do not return to their natal colonies after their first hibernation, and live solitarily and often hidden in tree cavities afterwards^48^. As a result, adult males were rarely found in the bat boxes and we could only obtain very few repeated measures for males. We used R (R 4.3.1, R Core Team, 2023^49^) to perform all our analyses, including estimation of measurement error and descriptive statistics. Our main aim was to discern species-specific from site-specific differences in fluctuating asymmetry along the 27 years of study. First, we ran a generalised linear model with mean asymmetry of all individuals as a response variable and the number of captures as a predictor variable. With this analysis, we wanted to check if the number of captures affect the extent of asymmetry. Next, we specified two-way mixed ANOVA models with forearm length as a response variable. The fixed term included the predictor variable side (two factors; left and right) to test for directional asymmetry. The random term included the predictor variable individual and the interaction of individual with side to test for fluctuating asymmetry^10^. Directional asymmetry was present in all species and could bias the analyses. Therefore, we subtracted half the mean asymmetry in *M. bechsteinii* (0.085/2), *M. nattereri* (0.036/2), and *P. auritus* (0.007/2) from the larger side and added half the mean asymmetry to the shorter side^50^. The measurement error was defined as the square root of the mean sum of squares (MS) of the residuals. In a second ANOVA, we included a random term with the interaction of the Observer ID and Side. With this additional ANOVA, we checked if Observer ID affects the direction of asymmetry. Since the observer had an effect on the side of asymmetry in *M. bechsteinii*, we did a posthoc tests with the emmeans package in R^51^. For each species separately, we looked at the distributions of the asymmetry values and did an Anscombe‐Glynn test to test for kurtosis and antisymmetry^10^. We specified general linear mixed-effect models with Gaussian error to investigate FA in the three bat species with the R package glmmTMB^52^. An alpha level of 0.05 defined statistical significance.

The response variable is asymmetry (continuous, ranging from 0 – 5.8 mm) with species (3-level categorical), year of birth (continuous), site (4-level categorical with BS [n _*M. bechsteinii*_ = 73; n _*P. auritus*_ = 103] as reference site) and their two-way interactions as fixed predictor variables. Additionally, individual ID and observer ID were random predictors because repeated measurements of any adult individual can indicate measurement error and different observers might vary in measurement precision. Different observers measured the bats during the course of the underlying long-term study^53^.

Using stepwise deletion, we selected the most parsimonious model and assessed AIC (Final model: AIC = -6731.2, Second to final model: AIC = -6728.3). The most parsimonious model excluded all two-way interactions. Outliers with an exceptionally high level of measurement error (> 0.5 mm difference between repeated measurements of the same individual) were removed after preliminary analyses. Since the model violated the normality assumption, with a skew toward small values, the final model had a Gamma error instead of a Gaussian error. We used mean [interquartile ranges] to compare FA between species and sites.

In a second model, we included *M. bechsteinii* only due to it being the only species occurring at all four sites. About 59% of all captured individuals were recaptured at least once, with an average of 4.8 recapture events (within or between years). We used all bats with at least three capture records (n = 519) to create three groups related to asymmetry, namely highly asymmetric (n _All_ = 15; n_*M. bechsteinii*_ = 12), symmetric (n _All_ = 48; n_*M. bechsteinii*_ = 37) and intermediate (n_All_ = 456, n_*M. bechsteinii*_ = 202). This grouping allowed us to investigate if highly asymmetric individuals have a shorter lifespan or lower lifetime reproductive success than all other individuals. Highly asymmetric individuals have a mean asymmetry of 0.4 mm across all measurements (which corresponds to ca. 1% of the total forearm length) and are consistently at least 0.3 mm longer or shorter on one side in the repeated measures, to account for the measurement error. For similar reasons, symmetric individuals according to our definition are consistently no more than 0.2 mm longer or shorter on the right or left forearm, and have a mean asymmetry of less than 0.05 mm across all measurements*.* Furthermore, bats with only three measurements must have at least one measurement showing no asymmetry to classify as symmetric. The intermediate group comprises individuals, who did not meet the criteria for the other two groups. Instead of discarding these individuals with unknown forearm asymmetry, we included them in the model. The three classes did not vary in the number of captures and thus, measurements (negative binomial glm, p = 0.49–0.59).

Since there were only two highly asymmetric *M. nattereri* and *P. auritus* individuals, the downstream analyses only included *M. bechsteinii.* The generalized linear models had age (continuous; gaussian) and reproductive output (count data, poisson) as response variables and symmetric (yes/intermediate/no), colony (categorical) and birth year (categorial) as predictor variables. To account for skewed data in the age model and overdispersed data in the reproductive output model, the final models had a Gamma and a negative binomial error, respectively.

## Supplementary Information

Supplementary Information.

## Data Availability

All the data used and generated in this study are available in this article’s supplementary information files.

## References

[1] Van Valen, L. A study of fluctuating asymmetry. *Evol.* **16**, 125–142 (1962).

[2] Parson, P. A. Fluctuating asymmetry: A biological monitor of environmental and genomic stress. *Hered.* **68**, 361–364 (1992). 10.1038/hdy.1992.51 1563968

[3] Mercola, M. & Levin, M. Left-right asymmetry determination in vertebrates. *Annu. Rev. Cell. Dev. Biol.* **17**, 779–805 (2001). 11687504 10.1146/annurev.cellbio.17.1.779

[4] Macleod, C. D. et al. Breaking symmetry: The marine environment, prey size, and the evolution of asymmetry in cetacean skulls. *Anat. Record.* **290**, 539–545 (2007). 10.1002/ar.20539 17516443

[5] Shine, R., Langkilde, T., Wall, M. & Mason, R. T. The fitness correlates of scalation asymmetry in garter snakes *Thamnophis sirtalis parietalis*. *Funct. Ecol.* **19**, 306–314 (2005).

[6] Tucić, B., Budečević, S., Manitašević Jovanović, S., Vuleta, A. & Klingenberg, C. P. Phenotypic plasticity in response to environmental heterogeneity contributes to fluctuating asymmetry in plants: First empirical evidence. *J. Evol. Biol.* **31**, 197–210 (2018). 29134739 10.1111/jeb.13207

[7] Dongen, S. V. Fluctuating asymmetry and developmental instability in evolutionary biology: Past, present and future. *J. Evol. Biol.* **19**, 1727–1743 (2006). 17040371 10.1111/j.1420-9101.2006.01175.x

[8] Koprivnikar, J. Interactions of environmental stressors impact survival and development of parasitized larval amphibians. *Ecol. Appl.* **20**, 2263–2272 (2010). 21265456 10.1890/09-1558.1

[9] Merlot, E., Quesnel, H. & Prunier, A. Prenatal stress, immunity and neonatal health in farm animal species. *Animal* **7**, 2016–2025 (2013). 23915487 10.1017/S175173111300147X

[10] Monteiro, L. R., Mellado, B., Nogueira, M. R. & de Morais-Jr, M. M. Individual asymmetry as a predictor of fitness in the bat *Carollia perspicillata*. *J. Evol. Biol.* **32**(11), 1207–1229 (2019). 31420901 10.1111/jeb.13522

[11] Wigley, B., Stillman, E., & Craig‐Atkins, E. Taking shape: A new geometric morphometric approach to quantifying dental fluctuating asymmetry and its application to the evaluation of developmental stress. *Archaeometry* 1–25 (2024).

[12] Anciães, M. & Marini, M. Â. The effects of fragmentation on fluctuating asymmetry in passerine birds of Brazilian tropical forests. *J. Appl. Ecol.* **37**, 1013–1028 (2000).

[13] Coda, J. A., Martínez, J. J., Steinmann, A. R., Priotto, J. W. & Gomez, M. D. Fluctuating asymmetry as an indicator of environmental stress in small mammals. *Mastozool. Neotrop.* **24**, 313–321 (2017).

[14] Gebremichael, G., Tsegaye, D., Bunnefeld, N., Zinner, D. & Atickem, A. Fluctuating asymmetry and feather growth bars as biomarkers to assess the habitat quality of shade coffee farming for avian diversity conservation. *R. Soc. Open Sci.* **6**, 190013 (2019). 31598226 10.1098/rsos.190013 PMC6731696

[15] Coda, J. A., Martínez, J. J., Serafini, V. N., Gomez, M. D. & Priotto, J. W. Phenotypic variability and developmental instability in rodents from different agricultural farming systems: Organic vs. conventional. *Mamm. Biol.* **101**(6), 1019–1032 (2021).

[16] Hopton, M. E., Cameron, G. N., Cramer, M. J., Polak, M. & Uetz, G. W. Live animal radiography to measure developmental instability in populations of small mammals after a natural disaster. *Ecol. Indic.* **9**, 883–891 (2009).

[17] De Coster, G. et al. Fluctuating asymmetry and environmental stress: Understanding the role of trait history. *PLoS ONE* **8**, e57966 (2013). 23472123 10.1371/journal.pone.0057966 PMC3589457

[18] Yosef, R., Kubicka, A. M., Brandsma, M. & Tryjanowski, P. A tale of two tails: Asymmetry in Great Grey Shrike (*Lanius excubitor*). *Avian Res.*10.1186/s40657-017-0094-1 (2018).

[19] Ueti, A., Pompeu, P. S., & Ferreira, R. L. Asymmetry compensation in a small vampire bat population in a cave: a case study in Brazil. *SB* **15**, 57–67 (2015).

[20] Manning, J. T. & Ockenden, L. Fluctuating asymmetry in racehorses. *Nature* **370**(6486), 185–186 (1994). 8028662 10.1038/370185a0

[21] Møller, A. P. & Swaddle, J. P. Asymmetry, developmental stability and evolution. (Oxford University Press, 1997).

[22] Beasley, D. A. E., Bonisoli-Alquati, A. & Mousseau, T. A. The use of fluctuating asymmetry as a measure of environmentally induced developmental instability: A meta-analysis. *Ecol. Indic.* **30**, 218–226 (2013).

[23] Cuervo, J. J., Dhaoui, M. & Espeso, G. Fluctuating asymmetry and blood parameters in three endangered gazelle species. *Mammal. Biol.* **76**, 498–505 (2011).

[24] Galeotti, P., Sacchi, R. & Vicario, V. Fluctuating asymmetry in body traits increases predation risks: Tawny owl selection against asymmetric woodmice. *Evol. Ecol.* **19**, 405–418 (2005).

[25] Voigt, C. C., Heckel, G. & Mayer, F. Sexual selection favours small and symmetric males in the polygynous greater sac-winged bat *Saccopteryx bilineata* (Emballonuridae, Chiroptera). *Behav. Ecol. Sociobiol.* **57**, 457–464 (2005).

[26] Dufour, K. W. & Weatherhead, P. J. Reproductive consequences of bilateral asymmetry for individual male red-winged blackbirds. *Behav. Ecol.* **9**, 232–242 (1998).

[27] Dufour, K. W. & Weatherhead, P. J. Bilateral symmetry and social dominance in captive male red-winged blackbirds. *Behav. Ecol. Sociobiol* **42**, 71–76 (1998).

[28] Gummer, D. L. & Brigham, R. M. Does fluctuating asymmetry reflect the importance of traits in little brown bats ( *Myotis lucifugus* )?. *Can. J. Zool.* **73**, 990–992 (1995).

[29] Breno, M., Bots, J. & Van Dongen, S. Heritabilities of directional asymmetry in the fore- and hindlimbs of rabbit fetuses. *PLoS ONE* **8**, e76358 (2013). 24130770 10.1371/journal.pone.0076358 PMC3794934

[30] Mundinger, C., Fleischer, T., Scheuerlein, A. & Kerth, G. Global warming leads to larger bats with a faster life history pace in the long-lived Bechstein’s bat (*Myotis bechsteinii*). *Commun. Biol.* **5**, 682 (2022). 35810175 10.1038/s42003-022-03611-6 PMC9271042

[31] Stapelfeldt, B. et al. Long-term field study reveals that warmer summers lead to larger and longer-lived females only in northern populations of Natterer’s bats. *Oecologia* **201**, 853–861 (2023). 36773071 10.1007/s00442-023-05318-9 PMC10038953

[32] Weidner, H. Unterschiedliche Unterarmlängen bei Fransen- (Myotis nattereri) und Bechsteinfledermaus (M. bechsteinii). *Nyctalus N.F.* **15**(1), 90 (2010).

[33] Weidner, H. Two further records of the Bechstein‘s bat (*Myotis bechsteinii* Kuhl, 1817) with different forearm lengths in a Thuringian reproduction colony. *Nyctalus (N.F.)* **20**(1), 154–56 (2022).

[34] Behr, O. et al. Territorial songs indicate male quality in the sac-winged bat *Saccopteryx bilineata* (Chiroptera, Emballonuridae). *Behav. Ecol.* **17**, 810–817 (2006).

[35] Dyrcz, A., Wink, M. & Kruszewicz, A. Male reproductive success is correlated with blood parasite levels and body condition in the promiscuous aquatic warbler (*Acrocephalus paludicola*). *The Auk* **2**(12), 558 (2005).

[36] Costanzo, A. et al. Lifetime reproductive success, selection on lifespan, and multiple sexual ornaments in male European barn swallows: Breeding success and selection in swallows. *Evol.* **71**, 2457–2468 (2017). 10.1111/evo.13312 28722759

[37] Cadée, N. Parent barn swallow fluctuating asymmetry and offspring quality. *J. Av. Biol.* **31**, 495–503 (2000).

[38] Stige, L. C., Slagsvold, T. & Vøllestad, L. A. Individual fluctuating asymmetry in pied flycatchers (*Ficedula hypoleuca*) persists across moults, but is not heritable and not related to fitness. *Evol. Ecol. Res.* **7**(3), 381–406 (2005).

[39] Swaddle, J. P. Within-individual changes in developmental stability affect flight performance. *Behav. Ecol.* **8**(6), 601–604 (1997).

[40] Culina, A., Linton, D. M. & Macdonald, D. W. Age, sex, and climate factors show different effects on survival of three different bat species in a woodland bat community. *GECCO* **12**, 263–271 (2017).

[41] Reusch, C. et al. The risk faced by the early bat: individual plasticity and mortality costs of the timing of spring departure after hibernation. *Oikos* **2023**, e09654 (2023).

[42] Kerth, G. & Reckardt, K. Information transfer about roosts in female Bechstein’s bats: An experimental field study. *Proc. R. Soc. B: Biol. Sci.* **270**, 511–515. 10.1098/rspb.2002.2267 (2003). 10.1098/rspb.2002.2267 PMC1691266 12641906

[43] Kerth, G. & Morf, L. Behavioural and genetic data suggest that Bechstein’s bats predominantly mate outside the breeding habitat. *Ethology* **110**, 987–999 (2004).

[44] Møller, A. P., Erritzøe, J. & Van Dongen, S. Body size, developmental instability, and climate change. *Evolution.* **72**(10), 2049–2056 (2018). 30095156 10.1111/evo.13570

[45] Mundinger, C., Van Schaik, J., Scheuerlein, A. & Kerth, G. Heat over heritability: Increasing body size in response to global warming is not stabilized by genetic effects in Bechstein’s bats. *Glob. Change Biol.* **29**, 4939–4948 (2023). 10.1111/gcb.16824 37340689

[46] Kerth, G. & van Schaik, J. Bechstein’s Bat *Myotis bechsteinii* (Kuhl, 1817) in *Handbook of the Mammals of Europe* (ed. K, Zachos, K.) 1–22 (Springer, 2022). 10.1007/978-3-319-65038-8_58-1.

[47] Kerth, G., Mayer, F. & Petit, E. Extreme sex-biased dispersal in the communally breeding, nonmigratory Bechstein’s bat (Myotis bechsteinii). *Mol. Ecol.* **1**(8), 1491–1498 (2002). 10.1046/j.1365-294x.2002.01528.x 12144668

[48] Scott, D. D. et al. Relatedness, parentage, and philopatry within a Natterer’s bat (Myotis nattereri ) maternity colony. *Pop. Ecol.* **60**, 361–370 (2018).

[49] R Core Team. R 4.3. 1: A Language and Environment for Statistical Computing https://cran.rstudio.com (2023).

[50] Palmer, A. R. Fluctuating asymmetry analyses: a primer in *Developmental instability: its origins and evolutionary implications: proceedings of the international conference on developmental instability: its origins and evolutionary implications* (Springer Netherlands, 1994).

[51] Lenth, R., Singmann, H., Love, J., Buerkner, P., & Herve, M. Emmeans: Estimated marginal means. *AKA least-squares means. ****1*****(7)**, (2018).

[52] Brooks, M. E. et al. glmmTMB Balances speed and flexibility among packages for zero-inflated generalized linear mixed modeling. *R J.* **9**, 378. 10.32614/rj-2017-066 (2017).

[53] Kerth, G. Long-term field studies in bat research: Importance for basic and applied research questions in animal behavior. *Behav. Ecol. Sociobiol.* **76**(6), 75 (2022). 35669868 10.1007/s00265-022-03180-y PMC9135593

