# Supplementary material for: Long-term patterns of forearm asymmetry in females of three syntopic bat species and its effects on individual fitness
Source: Sci Rep. 2024 Nov 20;14:28736. doi: 10.1038/s41598-024-80130-w (PMC11579011; doi:10.1038/s41598-024-80130-w)
Supplement: Supplementary file 1 — Supplementary Information. [file 41598_2024_80130_MOESM1_ESM.pdf]

Supporting information File 1

Long-term patterns of forearm asymmetry in females of three syntopic bat species and its effects on individual fitness

[1]Tobias Süess* and [1]Gerald Kerth

Table S1. Birth year but not asymmetry affects lifetime reproductive success in *M. bechsteini* (Negative binomial)

Predictor variable	Sample size	Estimate	SE	p-value
Intercept		1.48	0.27	< 0.001
Intermediate	202	-0.09	0.25	0.72
Symmetric	37	-0.28	0.26	0.28
Site GB2	104	0.14	0.13	0.27
Site HB	50	-0.03	0.16	0.86
Site UA	42	0.27	0.16	0.10
Birth Year 1996	1	0.59	0.58	0.3
Birth Year 1999	3	-0.30	0.45	0.46
Birth Year 2001	1	0.26	0.60	0.66
Birth Year 2003	1	0.19	0.65	0.78
Birth Year 2004	1	-1.75	1.1	0.10
Birth Year 2005	3	0.16	0.44	0.72
Birth Year 2006	63	0.19	0.32	0.54
Birth Year 2007	1	-0.51	0.82	0.54
Birth Year 2008	14	-0.63	0.38	0.10
Birth Year 2009	23	-0.39	0.34	0.26
Birth Year 2010	2	-0.11	0.54	0.83
Birth Year 2011	7	-1.31	0.56	0.02 *
Birth Year 2012	25	-0.46	0.34	0.19
Birth Year 2013	18	-0.88	0.43	0.04 *
Birth Year 2014	11	-0.63	0.38	0.10
Birth Year 2015	12	-0.66	0.41	0.10
Birth Year 2016	20	-0.32	0.35	0.35
Birth Year 2017	20	-0.90	0.45	0.04 *
Birth Year 2018	15	-1.52	0.80	0.06
Birth Year 2019	12	-1.44	0.80	0.06

Table S2. Birth year and colony but not asymmetry affects lifespan in *M. bechsteini* (Gamma)

Predictor variable	Sample size	Estimate	SE	p-value
Intercept		0.28	0.04	< 0.001*
Intermediate	202	-0.00	0.03	0.98
Symmetric	37	0.03	0.04	0.37
Site GB2	104	-0.09	0.03	< 0.001*
Site HB	50	-0.05	0.03	0.057
Site UA	42	-0.09	0.03	0.002*
Birth Year 1996	1	-0.13	0.03	0.06
Birth Year 1999	3	-0.04	0.06	0.51
Birth Year 2001	1	0.12	0.15	0.41
Birth Year 2003	1	-0.11	0.08	0.16
Birth Year 2004	1	0.23	0.19	0.22
Birth Year 2005	3	-0.007	0.06	0.91
Birth Year 2006	63	0.02	0.04	0.54
Birth Year 2007	1	-0.10	0.08	0.25
Birth Year 2008	14	0.11	0.05	0.04*
Birth Year 2009	23	0.10	0.05	0.03*
Birth Year 2010	2	0.03	0.08	0.68
Birth Year 2011	7	0.14	0.08	0.06
Birth Year 2012	25	0.04	0.04	0.30
Birth Year 2013	18	0.05	0.04	0.22
Birth Year 2014	11	0.05	0.05	0.35
Birth Year 2015	12	0.07	0.05	0.15
Birth Year 2016	20	0.35	0.07	< 0.001*
Birth Year 2017	20	0.15	0.05	0.003*
Birth Year 2018	15	0.16	0.05	0.005*
Birth Year 2019	12	0.25	0.08	0.004*

Table S3. Asymmetry is similar across species and birth years (Gamma).

Fixed effect	Sample Size	Estimate	SE	p-value
Intercept		6.73	0.63	< 0.001 ***
**Species *M. nattereri***	272	-0.39	0.45	0.390
**Species *P. auritus***	128	-0.50	0.45	0.2640
Site GB2	221	-0.88	0.39	0.025*
Site HB	143	-1.07	0.45	0.018*
Site UA	71	-0.90	0.46	0.050
Birth Year	-	-0.003	0.02	0.870
**Random effect**		**Variance**	**SD**	
Individual		3.28	1.81	
Observer		0.50	0.70	
